# Comparison of low-dose morphine intrathecal analgesia and sufentanil PCIA in elderly patients with hip fracture undergoing single spinal anesthesia — a randomized clinical trial

**DOI:** 10.1186/s12871-022-01677-7

**Published:** 2022-04-27

**Authors:** Zhifei Xu, Zairong Tang, Juan Yao, Dongliang Liang, Feng Jin, Ying Liu, Kai Guo, Xiulu Yang

**Affiliations:** Department of Anesthesiology, Gaoyou T.C.M. Hospital, 225600 Jiang Su, P. R. China

**Keywords:** Elderly, Hip arthroplasty, Intrathecal morphine, Low-dose, Spinal anesthesia

## Abstract

**Background:**

The complications of postoperative pain, such as hypertension, hypermetabolism, irritability, and postoperative cognitive dysfunction, significantly affect the postoperative rehabilitation of elderly patients. Intrathecal morphine prolongs analgesia after surgery, but has been implicated in nausea and vomiting, pruritus, postoperative respiratory depression, or apneic episodes. The present study explored the effect and safety of low-dose morphine used adjunctively with bupivacaine during single spinal anesthesia or sufentanil patient-controlled intravenous analgesia (PCIA) in elderly patients with hip fracture surgery. Since elderly patients often need anticoagulant therapy in the early postoperative period, single spinal anesthesia was involved in completing the operation in this study.

**Methods:**

Eighty elderly patients aged 70–85 years who underwent elective hip fracture surgery with single spinal anesthesia were divided into two groups, 12.5 mg of 0.5% hyperbaric bupivacaine with 100 µg of morphine (morphine group, group M) and 12.5 mg of 0.5% hyperbaric bupivacaine with 100 µg of sufentanil PCIA (sufentanil group, group S). The analgesia scores using the visual analogue scale (VAS), the Brinell comfort scale (BCS) were evaluated at 6, 12, 24, and 48 h after operation, and adverse reactions were recorded such as nausea and vomiting, pruritus, sedation, respiratory depression, and POD (postoperative delirium) with Delirium Rating Scale-r 98.

**Results:**

Within 24 h after operation, the analgesic and BCS scores of group M were better than those of group S (*P* < 0.05). Group M had higher frequency of skin pruritus than group S within 24 h, and the difference was statistically significant. The incidence of POD in group M (2 cases) was lower than that in group S (6 cases) (5.71% vs 18.18%) (*P* < *0.05*) with the DRS-r 98 scores. No significant difference was observed in nausea and vomiting between the two groups, and the difference of severe respiratory depression was not found in both groups.

**Conclusion:**

Compared with sufentanil PCIA, low-dose intrathecal morphine has a satisfactory analgesic effect, and little effect on the patient's cognitive function with low medical cost. Under effective respiratory monitoring, it can be used safely and effectively in elderly patients with hip fracture.

**Trial registration:**

Registered with the Chinese Clinical Trial Registry under ChiCTR2100042706. 26/01/2021.

## Background

Hip fractures, which are very common in elderly patients, may lead to deterioration of functional capabilities and activity level, as well as a greater risk of mortality [1, 2]. Rehabilitation of individuals after hip fracture is a global concern that has been gaining increased importance due to rising life expectancy worldwide [3]. Hip arthroplasty, including hemiarthroplasty and total hip replacement, is a common surgical procedure in elderly patients that can lead to severe postoperative pain, associated with increased cardiovascular negative consequences such as tachycardia and increased myocardial infarction oxygen consumption, and myocardial ischemia [4, 5]. Accidents and other adverse events may also disrupt the autoimmune system, affect sleep quality, and lead to a higher risk of postoperative delirium (POD) [6].

Inadequate pain control can prevent patients from performing the early functional exercise, thereby increasing their risk of thromboembolic disease and exacerbating infections, which in turn can lead to delayed discharge from the hospital and increased healthcare costs [7, 8]. Previous studies have demonstrated that more than 50% of elderly patients with total hip arthroplasty suffer from insufficient postoperative analgesia due to the side effects of opioids, resulting in varying degrees of chronic pain [9, 10]. Therefore, proper management of postoperative pain is necessary to improve the quality of outcomes in elderly patients and prevent postoperative complications.

In hip replacement surgery, effective postoperative analgesia and early functional exercise are the important parts of the Enhanced Recovery After Surgery (ERAS)[11]. At present, the commonly used analgesic methods in orthopedic surgery include intravenous opioids, local epidural anesthetics, peripheral nerve block, and local infiltration anesthesia [12]. Intravenous administration has the disadvantages of large dosage, insufficient analgesic effect, and may cause severe postoperative nausea and vomiting. As elderly patients often need early anticoagulant therapy and earlier functional exercise after the operation, continuous epidural analgesia was not used as the first choice in the present study. Herein, we aimed to investigate the effect and safety of low-dose morphine used adjunctively with bupivacaine during single spinal anesthesia in elderly patients undergoing hip replacement surgery.

## Methods

### General information

This single-center, double-blind, randomized study adopted a two-arm parallel-group design to validate the previously described analgesic effects of intrathecal morphine and sufentanil PCIA in hip arthroplasty in elderly patients. (Trial registry: ChiCTR2100042706, http://www.chictr.org.cn/listbycreater.aspx). All the conducted experiments were approved by the Ethics Committee of Gaoyou Traditional Chinese Medicine Hospital (Y20200526) and were performed in accordance with the Declaration of Helsinki (1964). Written informed consent was obtained from each patient of their relatives.

### Allocation and randomization

A total of 80 patients aged 70–85 years with hip fracture (ASA I-III) who received hip fracture surgery between January 2021 and April 2021 were included in this study. There were 72 patients with femoral neck fracture and 8 patients with intertrochanteric fracture. The exclusion criteria were: patients who were classified as ASA > III; had a BMI ≥ 35; had a history of allergic reactions and CNS-related disease and/or malignant hyperthermia; had a history of drug dependence or alcohol abuse; had the psychiatric and neurological disease; with long-term medicated with analgesics or tranquilizers; unable to complete the research process, or had the presence of severe respiratory or cardiovascular disease. However, patients with severe conditions who could tolerate surgery after adequate preoperative treatment and evaluation were not excluded from the study. As 6 patients did not meet the inclusion criteria, 74 patients were ultimately included in this study and divided into the intrathecal morphine group (group M, *n* = 38) and sufentanil PCIA group (group S, *n* = 36).

A computer-generated blocked randomization method was used to allocate subjects to the sufentanil PCIA or intrathecal morphine intervention. Random number tables generated the allocation sequence by the study coordinators, and the allocation was concealed in sealed envelopes. An anesthesia nurse, who was not otherwise involved in the study, prepared the drug after being given the randomization envelope by the coordinator. Patients were randomized to receive either 2 µg/kg sufentanil PCIA or 100 µg intrathecal morphine followed by 100 ml normal saline ‘PCIA’. The anesthesiologists received the study drug for the spinal procedure and were unaware of the treatment administered, as were the enrolled patients.

### Anesthesia procedures and treatment

On arrival to the operation room, electrocardiogram (ECG), noninvasive blood pressure (NIBP), heart rate (HR), and blood oxygen saturation (SpO_2_) were routinely monitored, and 3 L/min of oxygen was administered via an oxygen mask. Patients received single spinal anesthesia, which was administered with a pencil-point needle (25 gauge) inserted at the L_3_-_4_ or L _4_–_5_ interspace in the lateral position by midline approach or through lateral crypt [13]. Group M received 2.5 ml of 0.5% hyperbaric ropivacaine combined with 100 µg preservative-free morphine followed by 100 ml normal saline ‘PCIA’. Group S received 2.5 ml of 0.5% hyperbaric ropivacaine followed by 2 µg/kg sufentanil + 100 ml normal saline for PCIA after surgery. PCIA pump was started at 2 ml/h with a 2 ml bolus with 10-min lockout and with a 15 ml hourly maximum in the two groups. We used the VAS scores as the main outcome; the VAS and BCS scores in both groups were observed and recorded among elderly patients at 6, 12, 24, and 48 h after surgery. Adverse reactions such as pruritus, respiratory depression, postoperative delirium (POD), nausea, and vomiting were recorded in the two groups, respectively. For severe nausea and vomiting, subjects intravenously received 4 mg of ondansetron for antiemetic prophylaxis. During the recovery phase, pain (VAS score > 4 and subject request for additional analgesia) was treated with ketorolac 30 mg as needed to provide multimodal analgesia.

### Definitions

VAS was defined as mean movement pain score, which included straight leg raise, knee flexion, or hip flexion movement, when the affected limb was in the best functional position. VAS score classification was as follows: VAS = 0, no pain; VAS ≤ 4, mild pain, tolerable; VAS > 4–6, pain, tolerable; VAS 7–10, severe pain, intolerable. Respiratory depression was defined as breathing rate < 10 /min, and hypopnea was defined as a 30% decrease in the respiratory flow signal associated with a 3% decrease in oxygen saturation [14]. Sedation and BCS were scored as follows: grade 0, awake; grade 1, occasionally sleepy, easy to wake up; grade 2, frequent sleepiness, easy to wake up; grade 3, sleepiness, difficult to wake up [15, 16]. Cognitive function was assessed through the Delirium Rating Scale-r 98, which is based on 13 following items: sleep/wake cycle disturbance, perceptual disturbances and hallucinations, delusions, lability of effect, language, thought process abnormalities, motor agitation, motor retardation, orientation, attention, short-term memory, long-term memory, and visuospatial ability; the ratings for each item were 0 (no impairment), 1 (mild), 2 (moderate), and 3 (severe impairment). This is a valid and reliable measure for delirium severity that can be used clinically to monitor the course of illness when serially administered [17–19].

### Statistical methods

To determine the sample size required for our study, we performed a two samples independent *t*-test assuming homogenous variances. At the significance level of *α* = *0.05*, we anticipated and compensated for 5% attrition and planned to recruit 80 subjects. SPSS version 20.0 was used for statistical analysis. Data were expressed as mean ± standard deviation (SD). Student's independent *t*-test and repeated measurement analyses were conducted. Chi-square test and Fisher's exact test, whichever appropriate, were used to compare categorical variables. Repeated measures ANOVA was used to compare variables in both groups. Intervention state was considered as the between-subject factor, and time of evaluation as the within-subject factor. *P* < *0.05* was considered as statistical significance.

## Results

**Fig. 1:**
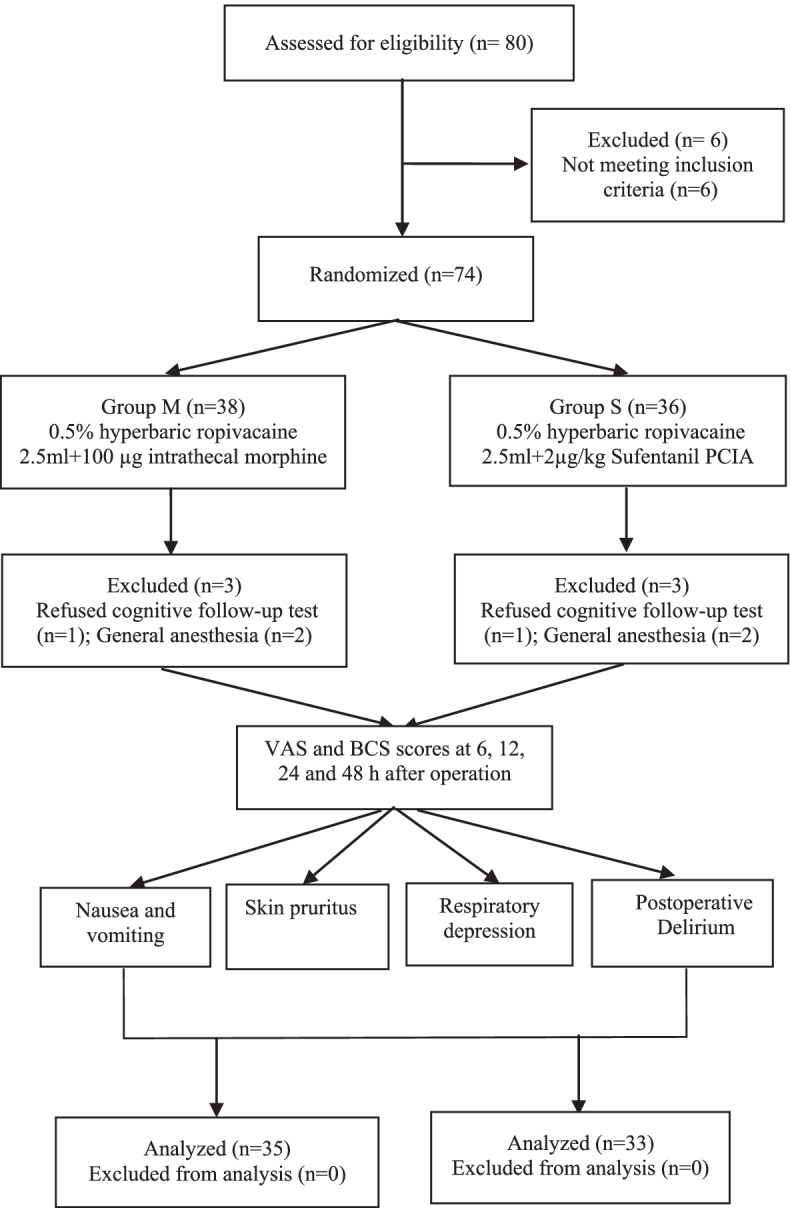
Consolidated Standards of Reporting Trials flowchart of patient recruitment

Among 80 patients included in the study, 6 were excluded for not meeting inclusion criteria. Among the remaining 74 patients, 2 refused to undergo postoperative neurological testing, and 4 patients who completed the operation underwent general anesthesia (Fig. 1). Finally, 35 patients in group M and 33 in group S were included in the analysis. The two groups of patients had similar demographic characteristics, and no difference was observed in the data between anesthesia and surgery (Table 1).

**Table 1:** Demographic and clinical characteristics ($\overline{x}$ ± s)

Admission characteristics	Group M (*n* = 38)	Group S (*n* = 36)
Age, (y)	75.2 ± 0.6	76.1 ± 0.7
Gender, (M/F)	26/12	25/11
BMI, (kg/m^2^)	28.6 ± 0.3	27.2 ± 0.2
MMSE score	26.8 ± 2.4	28.4 ± 2.1
Type Surgery, n (H/T)	28/10	24/12
Oxygen Saturation, (%)	95.9 ± 1.1	96.6 ± 1.2
Infusion volume, (ml)	952.6 ± 44.5	924.7 ± 52.2
Estimated blood loss, (ml)	212.8 ± 20.2	235.5 ± 15.8
Length of surgery, (min)	56.3 ± 1.3	59.7 ± 1.5
Duration of anesthesia, (min)	133.4 ± 12.6	145.5 ± 14.2

Data are given as mean ± SD

*M* Male, *F* Female, *H* hemi-arthroplasty, *T* total hip replacement

Within 24 h after operation, group M had a lower VAS pain score and higher sedation and BCS scores compared with group S. At 48 h after operation, group S had a higher VAS pain score and frequency of nausea and vomiting compared with group M; however, no significant difference was observed between groups (*P* > *0.05*). In addition, the number of patients with pruritus in group M was higher than that in group S, which occurred within 24 h after the operation. The DRS-r 98 scores in group M were significantly lower than those in group S within 48 h after operation; the incidence of POD in group S (6 cases) was higher than that in group M (2 cases) (all *P* < *0.05*; Tables 2 and 3). There was no significant respiratory depression in any of the groups (*P* > *0.05*).

**Table 2:** Comparison of VAS, BCS and DRS-r98 scores between the two groups ($\overline{x}$ ± s)

Time (h)	VAS score		BCS score		DRS-r 98 score	
	**Group M**	**Group S**	**Group M**	**Group S**	**Group M**	**Group S**
**6**	0.77 ± 0.12^▲^	1.18 ± 0.13	1.46 ± 0.09^▲^	0.88 ± 0.11	8.57 ± 0.18	9.12 ± 0.21
**12**	2.36 ± 0.12^▲^	3.29 ± 0.16	1.28 ± 0.07^▲^	0.97 ± 0.08	9.83 ± 0.17	10.12 ± 0.11
**24**	3.57 ± 0.13^▲^	4.03 ± 0.12	0.77 ± 0.08^▲^	0.52 ± 0.09	11.51 ± 0.23^▲^	13.55 ± 0.39
**48**	4.22 ± 0.08	4.30 ± 0.11	0.17 ± 0.06	0.33 ± 0.07	13.71 ± 0.35^▲^	17.09 ± 0.38

^▲^Compared with group S, *P* < *0.05*

**Table 3:** Comparison of adverse reactions between the two groups [n (%)]

Adverse reactions	Group M (*n* = 35)	Group S (*n* = 33)
Nausea/ vomiting	4 (11.43)	3 (9.09)
Pruritus	8 (22.86) ^▲^	2 (6.06)
Sedation/drowsiness	3 (8.57)	2 (6.06)
POD	2 (5.71) ^▲^	6 (18.18)
Respiratory depression	3 (8.57)	2 (6.06)

^▲^Compared with group S, *P* < *0.05*

## Discussion

Hip arthroplasty surgery is a major joint surgery commonly performed in elderly patients. Epidural analgesia is often administered during surgery as it offers excellent pain relief; nonetheless, it has been associated with various risks, including epidural hematoma, bradycardia, hypotension, and prolonged lower extremity motor block time. Therefore, functional exercise and effective postoperative analgesia are recommended after surgery for anticoagulation. In the present study, we used single spinal anesthesia with a low dose (100 µg) of morphine. Intrathecal morphine can provide effective analgesia for this operation and is often used as a component of multimodal analgesic regimens to facilitate early ambulation, rehabilitation, and ultimately improved recovery [20].

In the current study, the VAS scores of group M were significantly lower than that of group S at 24 h after surgery. At 48 h after operation, group S had a higher VAS pain score and frequency of nausea and vomiting compared with group M; however, no significant difference was observed between the two groups. This showed that intrathecal morphine has an exact analgesic effect, which is beneficial to early functional exercise and preventing or reducing complications such as lower extremity venous thrombosis in elderly patients. Although the number of patients with pruritus in group M was higher than that in group S within the first 24 h, this did not affect patients’ BCS scores after symptomatic treatment.

POD is one of the most prevalent perioperative complications of proximal femoral fracture surgery in elderly patients. It is associated with increased mortality, prolonged hospital stay, and impaired functional recovery and is prognostic of cognitive impairments and dementia [21, 22]. The factors influencing the incidence of POD during admission are pain and systemic opioid use [23]. In this study, the DRS-r 98 scores of group M were lower than that of group S; 2 cases of POD were observed in group M, and 6 cases were found in group S. According to existing studies, this might be due to the systemic effect of opioids, which possibly involve cognitive dysfunction, and intrathecal morphine that exerts a selective spinal effect with few systemic effects due to its hydrophilic nature [24, 25]. The present study showed that low-dose intrathecal morphine had an excellent analgesic and sedative effect without increasing the risk of POD in elderly patients, which is consistent with Koning’s research [26].

The main concern or complication of intrathecal morphine is delayed respiratory depression, especially in elderly patients. The incidence of respiratory depression of intrathecal morphine ranges from 0 to 9% [27]. This variability may be due to the different doses applied (25–500 µg) and the different definitions used for respiratory depression, such as reduced respiratory rate, decreased oxygen saturation, hypercapnia, naloxone administration, and increased sedation [28]. Previous studies have shown that 100 µg of intrathecal morphine can effectively relieve postoperative pain without increasing postoperative respiratory complications in patients with obstructive sleep apnea [29, 30]. In our study, a 100 µg dose of intrathecal morphine could provide an exact analgesic effect, although a few of patients had a slight decrease in blood oxygen saturation at 6–12 h after surgery; no severe respiratory depression was found in the patients from group M. Our results revealed that 100 µg intrathecal morphine could offer a reasonable compromise between analgesic efficacy and minimal side effects such as respiratory depression in elderly patients undergoing hip arthroplasty.

Although sufentanil has a good and sustained analgesic effects after surgery, the disadvantages of large dosage, insufficient analgesic effect, and intravenous opioids may lead to increased adverse reactions, such as nausea and vomiting, which may be risk factors affecting postoperative cognitive function in elderly patients. In addition, sufentanil PCIA requires special equipment and medical staff to follow up and adjust drug delivery dose according to the different degrees of analgesic effect of the patients after the operation, which significantly increases the workload of medical staff.

There are some limitations in the present study. First, we defined respiratory depression based on decreased ECG respiratory rate and fingertip pulse oxygen saturation, which can be affected by the patient’s voluntary activities, so arterial blood gas analysis should be used to accurately reflect the patient’s respiratory status. Second, the same dose of intrathecal morphine in patients with different BMI cannot accurately reflect the results of this study. Therefore, our results may not be applicable to populations with a higher BMI, and all of these data should be interpreted as exploratory and require further investigation.

In conclusion, compared with sufentanil PCIA, 100 µg of intrathecal morphine can provide effective postoperative analgesia without inducing serious adverse reactions, and it can reduce the impact of systemic opioids in elderly patients undergoing hip arthroplasty with single spinal anesthesia.

## Data Availability

The data that support the findings of this study are available from the corresponding author upon reasonable request.

## Abbreviations

PCIA: Patient-controlled intravenous analgesia
ChiCTR: Chinese Clinical Trial Registry
VAS: Visual analogue scale
BCS: Brinell comfort scale
POD: Postoperative delirium
DRS-r 98: Delirium Rating Scale-r 98
ASA: American Society of Anesthesiologists
CNS: Central Nervous System
BMI: Body mass index
ECG: Electrocardiogram
HR: Heart rate
NIBP: Non-invasive blood pressure
SpO_2_: Blood oxygen saturation
ANOVA: Variable one-way analysis of variance
